# Épidémiologie des accidents domestiques graves de l'enfant admis en réanimation pédiatrique polyvalente à l'hôpital d'enfants de Rabat-Maroc

**DOI:** 10.11604/pamj.2015.20.28.5760

**Published:** 2015-01-12

**Authors:** Mostafa Rafai, Nour Mekaoui, Naoufal Chouaib, Hicham Bakkali, Lahcen Belyamani, Alae El Koraichi, Salma Ech-Cherif El Kettani

**Affiliations:** 1 Service des Urgences Médico-chirurgicales de l'Hôpital Militaire d'Instruction Mohammed V, CHU Ibn Sina, Rabat, Maroc; 2 Service de Réanimation Pédiatrique Polyvalente, Hôpital d'Enfants de Rabat, CHU Ibn Sina, Rabat, Maroc; 3 Faculté de Médecine et de Pharmacie, Université Mohammed V, Rabat, Maroc

**Keywords:** Enfant, accident domestique grave, prévalence, mortalité, prévention, child, severe domestic accident, prevalence, mortality, prevention

## Abstract

**Introduction:**

Les accidents domestiques de l'enfant représentent un vrai problème de santé publique dans les pays industrialisés. Au Maroc, la priorité en santé publique est toujours donnée aux pathologies infectieuses, et bien qu'elle soit très peu décrite, la pathologie accidentelle de l'enfant devient de plus en plus fréquente dans notre pays avec une mortalité importante. L'objectif est de mettre le point sur la prévalence, la gravité, les aspects étiologiques, les facteurs de risque et les circonstances de survenue de ces accidents, ainsi que les moyens de prévention active et passive.

**Méthodes:**

Enquête rétrospective descriptive sur une période de douze mois portant sur tous les enfants pris en charge pour accident domestique grave au service de réanimation pédiatrique polyvalente de l'hôpital universitaire d'enfants de Rabat.

**Résultats:**

Parmi 698 admissions, 108 cas d'accidents domestiques graves ont étaient colligés (soit 15,5%), L’âge moyen des enfants était de 04ans tout accident confondu, avec un sex-ratio de 1,08 en faveur des garçons. L’évolution générale était marquée par le décès de 16 enfants (soit 14,8%) parmi 164 décès toute pathologie confondue au cours de la même période d’étude (soit 9,75% des décès) avec une durée moyenne d'hospitalisation de 04jours. les brûlures constituaient le premier accident dans notre série par 37cas, et elles étaient la première cause de mortalité par huit cas; par ailleurs, la population la plus à risque de brûlure était les nourrissons (67,6%). L'inhalation intrabronchique d’épingle à foulard (accident particulier dans notre contexte islamique) à été retrouvée chez six cas.

**Conclusion:**

Les accidents domestiques de l'enfant constituent rarement une préoccupation de premier plan dans la population alors qu'ils sont parfois très graves et source d'une mortalité importante. Le meilleur traitement reste la prévention active et passive.

## Introduction

Pour l´OMS (Organisation Mondiale de la Santé) l´accident se définit par “un événement qui, dans une séquence chronologique généralement courte, conduit à un transfert d´énergie entre une source et une structure cible susceptible d´être modifiée, de manière réversible ou non”. Les accidents domestiques sont définis comme “les accidents qui surviennent à la maison ou dans ses environs immédiats” [1]. Les accidents domestiques de l'enfant représentent un problème de santé publique dans les pays industrialisés et font l'objet de nombreuses campagnes de prévention [2]. Au Maroc, la priorité en santé publique est toujours donnée aux pathologies infectieuses, et bien qu'elle soit très peu décrite, la pathologie accidentelle devient de plus en plus fréquente dans notre pays avec une mortalité très importante. **L'objectif principal** de ce travail avait pour objectif t de mettre le point sur la prévalence, la gravité et les aspects préventifs de ces accidents toujours sous-estimés au Maroc en ce basant sur les données épidémiologiques d'un service de réanimation pédiatrique Marocain, afin d'optimiser les mesures de prévention et contribuer à l’élaboration de recommandations et de programmes nationales pour la sécurisation de l'environnement domestique de l'enfant, en initiant ce genre d'enquête dans notre contexte où les données épidémiologiques restent très insuffisantes et non actualisées. **Les objectifs secondaires**: soulever les facteurs de risque et circonstances de survenue, Discuter les moyens de prévention à la fois active et passive décrites dans la littérature, et en Proposer d'autres, Rappeler et décrire les circonstances et particularités épidémiologiques d'un accident domestique particulièrement fréquent et spécifique dans notre contexte de pays islamique: l'inhalation intrabronchique d’épingle à foulard, Indiquer l'utilité de ce genre de travaux dans l'enrichissement des sources d'information et dans la prévention de ces accidents fréquents et potentiellement graves.

## Méthodes

Il s'agissait d'une étude rétrospective descriptive sur une période de douze mois, portant sur tous les enfants pris en charge pour accident domestique grave au service de réanimation pédiatrique polyvalente de l'hôpital universitaire d'enfants de Rabat/Maroc. L'aval du comité d’éthique n'a pas été nécessaire vu le caractère rétrospectif et strictement descriptif de l’étude. Le diagnostic d'accident domestique se faisait sur la base des définitions de l'OMS et des sociétés savantes dans le domaine; quant à l'admission en réanimation, elle se faisait sur la base de critères reconnus, communs, bien définis et recommandés dans la littérature pour chaque étiologie (le caractère accidentel domestique des lésions ne constitue en aucun cas un critère de gravité pour admission en unité de soins intensifs). L'enregistrement des enfants se faisait à mesure de leur admission au service de réanimation en provenance des services des urgences ou d'un autre service d'hospitalisation pour prise en charge d'accident domestique grave nécessitant une prise en charge réanimation. Les enfants victimes d'accidents domestiques n'ayant pas nécessité l'admission en réanimation ont été exclus de l’étude et aucun autre critère d'exclusion n'a été adopté. Pour tous les enfants inclus, les données générales suivantes ont été recueillies: l’âge, le sexe, l'indication d'admission en réanimation, la durée d'hospitalisation, l’évolution et le type d'accident avec des renseignements spécifiques pour chacun (Brûlures: degré, superficie et localisation + agent causal; Corps étrangers: nature + siège; Intoxications: nature de la substance toxique en cause; Chutes: lieu et sa hauteur; Noyades: par eau douce ou salé + lieu).

## Résultats

### Données générales tous accidents confondus

**Figure 1:**
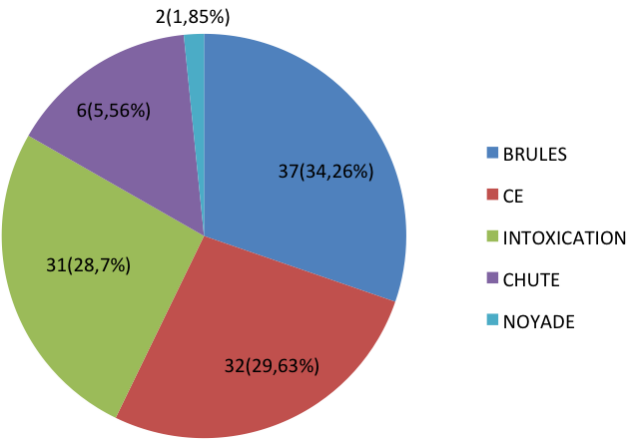
Principales étiologies retrouvées

Au cours de la période d’étude, 108 cas d'accidents domestiques graves ont été colligés parmi 698 admissions en réanimation pédiatrique toute pathologie confondue (soit **15,5% des admissions**). L’âge moyen des enfants était de 04ans (extrêmes 03mois-15ans), avec un sex-ratio de 1,08 (56 garçons contre 52 filles), l’évolution était marquée par le décès dans 16 cas(soit **14,8%**) parmi 164 décès toute pathologie confondue au cours de la même période d’étude (soit **9,75% des décès**) avec une durée moyenne d'hospitalisation de 04jours (extrêmes 1-19jours);les principales étiologies (Figure 1) étaient les brûlures, les intoxications, les corps étrangers, les chutes et les noyades.

### Données générales et spécifiques par type d'accident

#### Données générales démographiques

Les brûlures représentaient la première étiologie des accidents domestiques et la première cause de mortalité, suivies des intoxications; les données générales démographiques détaillées par type d'accident sont présentées dans le (Tableau 1).

**Tableau 1:** Variables démographiques de la population en fonction de l’étiologie

Accident	N(%)	Age Moyen (extrêmes)	Sex-ratio
Brûlures	37(34,26)	16 mois (03-14ans)	1,05(19M/18F)
Intoxications	31(28,7)	7 ans (1-15ans)	1,06(16M/15F)
Corps Etrangers			
-Intra-œsophagiens	18(16,67)	4 ans (6 mois- 4ans ½)	1,1(9M/8F)
-Intra-trachéaux (Sd de pénétration)	14(12,96)	4 ½ ans (1-15ans)	1,3(8M/6F)
Chutes	06(5,56)	6 ans (2-15ans)	1(3M/3F)
Noyades	02(1,85)	3 ans (pour les deux cas)	1(1M/1F)

#### Données spécifiques

**Figure 2:**
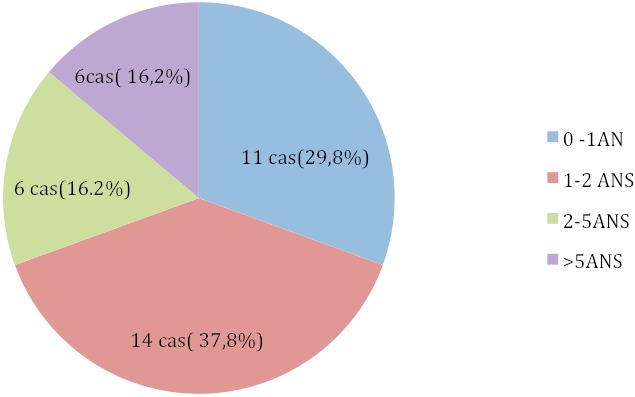
Répartition des brûlures selon la tranche d’âge

Pour les brûlures (37cas), on comptait 36cas par liquides chauds (eau bouillant dans la majorité des cas) et un seul cas d’électrisation; La répartition selon la tranche d’âge (Figure 2) trouvait: 11cas (0-1an), 14cas (1-2ans), 6cas (2-5ans), 6cas (≥5ans). les cas de brûlures par liquides chauds associaient tous des zones de deuxième degré profond et d'autres de troisième degré en même temps, avec des superficies brulées qui variaient entre 18% et 79% de surface corporelle selon les tables de Lund et Browder et l'indication d'admission à été faite chez 34cas sur la base d'une surface brulée ≥ 20%(10% chez le nourrisson) +/- associée à d'autres critères de gravités, les 2 autres cas ont été admis secondairement dans un tableau de sepsis sévère après une admission initiale dans un service de chirurgie.

Pour les intoxications (31cas) (Tableau 2), deuxième étiologie et deuxième cause de mortalité, on trouvait: les médicaments en premier lieu par 10cas, suivies des pesticides organophosphorés par 7cas, les raticides par 3cas, les produits ménagers (caustiques) par 2cas puis les d'origine indéterminée (dont les plantes) par 9cas; les intoxications par ingestion de substances caustiques ont été admis dans un tableau d'ingestion massive’ 150ml +/- associé à des troubles de conscience, pour les autres intoxications médicamenteuses, par les pesticides, les raticides ou indéterminées, ont été admis soit parce qu'elles étaient symptomatiques(présence d'une défaillance circulatoire, respiratoire ou neurologique, ou présence d'un syndrome toxique typique ou spécifique de la substance) soit asymptomatiques(devant le caractère imprévisible de l’évolution de certaines intoxications ou l'origine indéterminée d'autres pour surveillance-monitorage rapprochée à la phase initiale).

**Tableau 2:** Produits incriminés dans les intoxications

Type	Nombre (%)
médicamenteux	10(32,25)
Pesticides (organophosphorés)	7(22,6)
Raticides	3(9,7)
Produits ménagers	2(6,45)
Inconnue (dont les plantes)	9(29)

Les corps étrangers(32cas) étaient soit intra-œsophagiens par 18cas, soit intra-trachéaux(syndrome de pénétration) par 14cas, et les objets en cause étaient surtout: les pièces de monnaie, les cacahuètes, et les épingles à foulard chez les jeunes filles de 10 à 15ans qui portent le voile islamique(6cas, tous au niveau des voies aériennes); l'admission en unité de soins intensifs était systématique même pour l'extraction de corps étrangers intra-œsophagiens asymptomatiques sans indication d'hospitalisation” (vue que c'est le service référent chargé de la prise en charge des deux types de corps étrangers dans tout l'hôpital). Quant aux chutes (6cas), on notait 4 cas de chutes d'escaliers et les 2 autres cas de balcons; les 6 cas ont été admis en raison de la présence d'un traumatisme crânien modéré ou grave, isolé ou associé dans le cadre d'un polytraumatisme(le cas des deux chutes de balcons). En fin, les 2cas de noyades étaient tous les deux suite à une chute dans un puits (eau douce); les deux cas étaient admis dans un tableau de grand hypoxique-anoxique avec troubles de consciences et détresse respiratoire.

#### L’évolution

Dont les paramètres (durée d'hospitalisation et décès) détaillées en fonction de ’étiologie sont relatées dans le (Tableau 3).

**Tableau 3:** Évolution en fonction de l’étiologie

Accident	Durée moyenne d'hospitalisation (extrêmes)	Nombre de Décès
Brûlures	7 jours (1-18jours)	8
Intoxications	2,5 jours (qlq heures-4jours)	5
Corps Etrangers		
-Intra-œsophagiens	8 heures (qlq heures-24heures)	0
-Intra-trachéaux(Sd de pénétration)	2 jours (qlq heures-9jours)	1
Chutes	3 jours (2-5jours)	1
Noyades	5 jours (1-9jours)	1

## Discussion

Les accidents domestiques faisant partie des accidents de la vie courante(AcVC), sont définis comme “des événements fortuits, dommageables survenant brutalement au domicile des victimes ou des environnants immédiats”; Très variés, ils sont caractérisés par leur fréquence et leur bénignité habituelle, même si certains peuvent entraîner des séquelles graves, voire la mort.ils concernent principalement les intoxications, les chutes, les brûlures, les électrisations, les noyades, l'ingestion de corps étrangers et les morsures d'animaux [1], bien que de nouveaux accidents domestiques émergents ont été décrits récemment par Claudet en 2010 [3].

On peut classer les accidents domestiques en trois catégories: accident non grave, ne mettant en jeu ni le pronostic vital ni fonctionnel (prise en charge en ambulatoire ou dans un service de médecine), accident grave mettant en jeu le pronostic fonctionnel seul (prise en charge dans un service de chirurgie), accident grave engageant en moins le pronostic vital (prise en charge essentiellement en réanimation).

Les données épidémiologiques concernant les accidents domestiques de l´enfant ne peuvent être valablement considérées comme le reflet du phénomène accidentel parce qu´elles ne représentent qu´une petite partie du problème [4], en faite ces accidents se produisent toujours dans l'indifférence quasi généralisée, tant des autorités de santé que du grand public dans notre cotexte de pays en voie de développement où l'intérêt en matière de pathologie accidentelle est donné surtout aux accidents de la voie publique. Et pourtant, on assiste à une augmentation très importante de la prévalence de ces accidents qui constituent un sérieux problème de santé publique dans le monde entiers, c'est ainsi que sur le plan européen, le programme EHLASS, vise depuis 1981 à établir un système harmonieux pour le recueil et l´échange des données sur les AcVC [5]. Au Maroc la documentation et la littérature concernant les aspects épidémiologiques, évolutifs et préventifs des accidents domestiques de l'enfant sont très pauvres, ce qui contribue significativement à l'insuffisance profonde constatée en sources d'informations. Sur une année, nous avons pu colliger 108 cas d'accidents domestiques graves sur 698 admissions (soit 15,5%) au service de réanimation pédiatrique de l'hôpital d'enfants de Rabat(Maroc) avec 16décès (soit 14,8% des accidents domestiques); la prévalence et la mortalité des accidents domestiques de l'enfant varient considérablement d'un pays à l'autre[5]. selon l'enquête permanente sur les accidents de la vie courante 2000-2003 en France, on estime, que les moins de 15ans sont victimes chaque année de 1,5 à 2 millions d'AcVC avec recours aux urgences hospitalières [6]. En Afrique, l'incidence est également élevée: 381cas en l'an 2000 en Algérie et 1800 cas en un an à l'hôpital de Sfax en Tunisie [5, 7], pour Ka et al. [8] au Sénégal, la pathologie accidentelle constitue 3% des causes d'hospitalisation des enfants de 0 à 15ans, quant à Mbika-Cardorelle et al. [9], ils rapportent une fréquence de 2,8% des admissions dans l'unité de soins intensifs pédiatrique du CHU de Brazzaville (Congo).

L’âge moyen de nos patients (48 mois), était comparable à celui de Mbika-Cardorelle et al [9]. Au Congo en 2003(42 mois). Le jeune âge de la plupart des victimes d'accidents domestiques dans notre contexte tout comme ailleurs pourrait s'expliquer par le fait que les enfants de jeune âge sont difficiles à surveiller a cause de l’éveil de leur curiosité et parce qu'ils passent plus de temps à la maison [4], c'est ainsi qu'avant 6 ans, le champ visuel de l'enfant est restreint latéralement, il ne fait pas la différence entre voir et être vu, estime mal les distances, n'appréhende pas le vide, localise mal l'origine des bruits qui l'entourent, ne fait attention qu’à une chose à la fois, ne peut coordonner plusieurs mouvements [10]. Le sex-ratio général de notre population était de 1,08 en faveur des garçons, et en l'analysant par type d'accident, on le trouve toujours ≥1 en faveur des garçons; en France entre 2000-2003, on trouvait un sex-ratio garçon/fille de 1,4 [6]. Cette prédominance masculine à été rapporté dans plusieurs travaux dans d'autres pays [5, 11], et qui peut être expliquée par la turbulence des jeunes garçons [4].

Par ailleurs, nos principales étiologies retrouvées étaient par ordre de fréquence décroissante: les brûlures, les corps étrangers, les intoxications, les chutes puis les noyades. Et ce sont les principaux accidents domestiques classiques décrits dans la littérature [1, 4, 6, 9, 12–14]. En France la chute est le mécanisme le plus fréquent: 55% [11]; Claudet en 2010 [3] à mis le point sur de nouveaux accidents domestiques qui émergent par mésusage de certains produits, détournement de la fonction initiale d'autres, modes socioculturelles ou décoratives = risques liées à l'ameublement et le mobilier, à l'utilisation de siège-auto, à la décoration, aux mini-aimants, aux nouveaux animaux de compagnie [3]. Les brûlures constituaient le premier accident dans notre série. D´après les conclusions du système européen de surveillance des accidents, les brûlures sont des accidents qui surviennent une fois sur cinq chez les enfants de moins de 5 ans, dans notre série 83,8% des brûlés avaient moins de 5ans (soit quatre enfants sur cinq). Les brûlures par liquides chauds (projection, immersion) sont de loin les plus fréquentes, Il s´agit en général d´une projection de liquide chaud contenant des liquides variés, et les nourrissons qui deviennent autonomes en sont les principales victimes (queue de casserole, rebord de récipient attiré à soi, bol renversé…) [4], 67,6% des brulés dans notre série étaient des nourrissons (population la plus à risque de brûlure et la plus vulnérable). Les inhalations de corps étrangers au niveau des voies aériennes sont dues une fois sur deux à des cacahuètes, viennent ensuite les autres corps végétaux (pistache, noix, noisette, amandes,) dans 20 à 25% des cas, puis les objets de petite taille (pièces de monnaie, jetons, billes,) [15]. La nature des CE est influencée par les conditions socioculturelles et régionales. Dans le monde islamique, des épingles métalliques droites sont largement utilisées pour fixer le foulard autour de la tête chez les jeunes filles qui commencent à porter le voile islamique à partir de l’âge de 10 à 15 ans. Il s'agit d’épingles métalliques longues de 2 à 3 cm avec une tête en perle de plastique, radiotransparente, et une extrémité métallique pointue [16–18]. En pensant tenir fermement l’épingle entre les lèvres lors de la mise en place du foulard, l’épingle peut être facilement inhalée en parlant, riant ou en toussant. Des études ont été menées ces dernières années pour mettre le point sur ce corps étranger particulier dans le monde islamique et illustrer les circonstances et les conséquences lourdes de son inhalation chez les femmes et les jeunes filles [17, 18], mais très peu sont les études qui ont été consacrés aux particularités de ce corps étranger chez l'enfant; c'est ainsi que El Koraichi et al. [16] en 2011 ont décrit les aspects épidémiologiques, cliniques, thérapeutiques et évolutifs d'inhalation d’épingle à foulard au niveau des voies aériennes chez les jeunes filles, sur une série de 36 cas à l'hôpital d'enfant de Rabat, Maroc; quant aux corps étrangers de la sphère digestive, ils sont le plus souvent sans conséquence et rares sont les cas graves qui nécessitent une ablation sous anesthésie générale (2%) [19].

Les intoxications accidentelles peuvent être d´origines extrêmement variées (médicaments, produits caustiques, raticides, plantes, etc.), une diversité qui conduit à des tableaux cliniques aussi variés et multiples. Elles concernent encore une fois plus souvent les garçons que les filles (sex-ratio de 1,06 en faveur des garçons dans notre série), et le pic de fréquence concerne la tranche d´âge 1-4 ans [1, 4]. En France, on estime les intoxications accidentelles à 110 000 cas par an tout âge confondu, au Québec les statistiques rapportent 49 650 cas par an [20]. Ces intoxications sont plus fréquentes entre 11 heures et 14 heures et entre 19 heures et 20 heures [1]. La chute est de loin la cause la plus fréquente d'accidents domestiques dans la littérature (55% des AcVC et 73% avant 1 an en France) [13]. La tolérance des parties molles de l'enfant et la plasticité de son squelette font que la majorité de ces chutes sont sans grandes conséquences. Avant l’âge de 1 an, la réception se fait presque toujours sur le crâne (80% des cas) [21, 22]; d'après l'OMS, les chutes mortelles sont essentiellement dues aux défenestrations, chutes de balcons, de toit, d'escaliers et de lits superposées. Cependant, les défenestrations sont responsables de 20% de décès immédiat et 25% de handicaps graves [4]. Quant à la noyade, c'est la première cause de mortalité par accident domestique chez l'enfant en France (0,7 décès pour 100 000 enfants) [13] avec un pic entre 1 à 4 ans [4]. Les maladies cardiologiques à risque de syncope sont reconnues comme facteurs de risque de noyade chez l´enfant [4].

9,75% des décès toute pathologie confondue étaient par accidents domestiques dans notre série au cours de la même période d’étude; En 2006,266 enfants de moins de 15ans sont décédés d'AcVC en France métropolitaine, soit un taux de 2,4 décès pour 100 000(4). Suprano et al. [4] en 2003 ont conclu aux résultats suivants: 1 décès sur 5 entre 1 et 4 ans; 1 décès sur 8 entre 5 et 14 ans; en faite c'est dans la classe d’âge, de 1 à 4 ans, que la mortalité par accidents domestiques est la plus importante, évaluée par l´OMS à 33/100 000 habitants en 1993 pour l´ensemble des pays membres de l´Union européenne. Il est à noter par ailleurs que le taux de mortalité par accident est plus important chez les garçons que chez les filles [4]. Dans notre série la première cause de mortalité était les brûlures (7/15 décès), ce qui concorde avec les résultats Mbika-Cardorelle [9] en 2003 avec 4 décès/8 et ceux de Mabiala-Babela [12] en 2010 avec 2 décès /4, par ailleurs Thélot [13] en 2010 rapporte la noyade comme 1^ère^ cause de mortalité par 80/266 décès par AcVC en France métropolitaine. La protection de l'enfant contre les risques domestiques passe tout d'abord par une meilleure connaissance des facteurs de risque et circonstances de survenue. C'est ainsi, et pour conduire une politique de prévention des accidents à la fois adaptée et efficace, deux conditions essentielles sont donc à réunir [23, 24]: une connaissance actualisée des risques, grâce à un recueil permanent de données, accompagné d'une exploitation rapide et d'une diffusion large de ces résultats; un travail pluri-institutionnel.

D'une façon générale, on distingue les facteurs endogènes liés à l'enfant lui-même et les facteurs exogènes liés à son environnement matériel et psycho-social. Cependant Les caractéristiques physiologiques et psychologiques de l´enfant en font un être particulièrement vulnérable. Sa petite taille, son immaturité sensorielle, sa coordination psychomotrice imparfaite l´exposent à des risques particuliers [2]; La période du « tout à la bouche » entre 8 mois et deux ans, moyen pour porter à sa connaissance les objets autour de lui, est particulièrement critique pour les intoxications accidentelles et l´inhalation de corps étrangers. Chez le petit enfant, l´important volume de la tête par rapport à l´ensemble de son corps et à l´apparition de la marche, entraîne de plus fréquentes chutes, le plus souvent en avant [4]; Le jeune âge de la mère (moins de 20 ans), l'absence de l'affectivité familiale, une structure et un fonctionnement familial atypiques (abus de substances toxiques, les familles très nombreuses, monoparentales ou recomposées,..), le niveau socio-économique bas, le niveau culturel modeste, l'activité professionnelle importante des parents, tous éléments ont font les principaux facteurs exogènes psychosociaux [25], rappelant aussi que les parents sous-estiment souvent les capacités motrices et la curiosité de leurs enfants, et surestiment leur capacité de se souvenir des instructions de sécurité et de les appliquer [10]; reste les facteurs de l'environnement matériel de l'enfant représentés essentiellement par les éléments structuraux des logements, le mobilier, les articles de jeux et de puériculture non conformes, les produits dangereux à savoir les médicaments laissés à la portée des enfants, les puits non protégés dans les campagnes,..Etc. [4].

La réflexion sur les accidents domestiques est pluridisciplinaire mais aussi inter sectorielle. Elle fait appel à des démarches structurelles et organisationnelles (mesures réglementaires et législatives, politique d'aménagement de la ville, d’équipements,…) mais aussi informatives et éducatives, d'une très grande diversité en termes de compétences requises [24]. Ces démarches et stratégies de prévention peuvent être classées en prévention active et passive; on ce qui concerne la prévention active, il s'agit essentiellement de l’éducation des parents, et de celle des enfants après 6-8 ans[10], ces moyens d’éducation sont d'abord les campagnes d'information et de prévention sous forme de visites à domicile d'infirmières-puéricultrices, les outils pédagogiques sous forme de livres, brochures, vidéos, jeux, etc., puis les réunions d'information proposées par les crèches et les haltes garderies[10, 26]. Cependant les parents ne sont pas en mesure d'assurer à eux seuls cette sécurisation, c'est ainsi qu'apparait le rôle primordiale de la prévention passive sous forme de mesures législatives et de réglementation par la mise en place d'une législation pour la sécurité des produits, d'une législation destinée à assurer la protection des enfants, etc., ces mesures de prévention passive semblent être les plus efficaces, car elles touchent d´emblée un grand nombre d´individus et ne dépendent pas de paramètres individuels [4]. L'OMS s'inscrit dans ce cadre, à travers certains de ces programmes visant la promotion de la sécurité (safe community) [24]. Ces stratégies de prévention active et passive se complètent et les mesures législatives ne servent à rien si elles ne sont pas respectées par les parents[10], ce qui à été prouvé par une étude publié par Hue [27] en 2011 et portant sur 3 situations (les chutes dans les escaliers par des barrières de sécurité, les chutes des sièges par des harnais et les intoxications accidentelles par le mode de rangement et les emballages de sécurité), qui montrait que la majeure partie de ces 3 types d'accidents observés aux urgences en France est liée à l'absence d'application d'un moyen de prévention par les familles et non à un échec de ce moyen). Sznajder et al. [14] ont montré que la distribution à titre gracieux de trousses de prévention, accompagnée de conseils simples à adopter, permet à des familles souvent défavorisées de modifier leur comportement et d'aménager leur appartement en vue de plus de sécurité.

En faite, et malgré leur importance, les accidents domestiquent en France n'occupent que la 9^ème^ position dans les craintes de risques pour la santé chez les 12-25 ans, selon les enquêtes du baromètre santé de l'institut national de prévention et d’éducation pour la santé [28], ce manque d'intérêt est due essentiellement à un obstacle sémantique, c'est que l'accident est considéré comme inévitable parce que justement c'est un accident, et on se réfugie ici dans l'acceptation d'une sorte de fatalité [24]. Autres obstacles à la prévention sont à soulever, au Maroc par exemple, la priorité est toujours donnée à la pathologie infectieuse, alors qu'on matière de pathologie accidentelle de l'enfant on ne dispose pas encore d'un système de prévention primaire; la carence en connaissances épidémiologiques sur ces accidents, la pauvreté en sources d'information et le manque de stratégies de prévention adaptés aux populations les plus vulnérables [4] ont font des obstacles universels notamment dans les pays pauvres ou en voie de développement. Les sources d'information en matière de pathologie accidentelle de l'enfant doivent être hétérogènes, et des études spécifiques devraient être entreprises pour préciser, dans chaque groupe de cas, les circonstances précises de survenue: quelles étapes ont conduit à l'accident, dans quel contexte de risque se trouvait l'enfant avant l'accident, etc. [29]. Selon Lawrence [30], environ le tiers des décès par accidents chez les enfants serait évitable avec les seuls moyens et ressources disponibles. Nous proposons à travers ce travail, d'autres mesures de prévention active et passive, autres que celles rapportées dans la littérature; cependant et pour enrichir et diversifier les sources d'information, nous recommandant: *Primo*, l’élaboration de fiches simples et faciles à remplir par les praticiens pédiatres, chirurgiens pédiatres, urgentistes, réanimateurs, généralistes, etc., chaque fois qu'ils sont devant à un accident domestique de l'enfant, et les envoyer systématiquement et périodiquement au ministère de la santé(comme c'est le cas pour certaines pathologies infectieuses à déclaration obligatoire par exemple). Telles fiches doivent comporter des renseignements précis et ciblés sur l'enfant lui-même, son environnement matériel et psycho-social, l’évolution, et ses coordonnées pour le suivi d’éventuelles complications d'ordre fonctionnelles (handicaps). *Secundo*, indiquer la mention “accident domestique” dans les certificats de décès des enfants décédés par ces accidents. *Tertio*, pour les enfants hospitalisés dans une structure hospitalière ou même suivis à titre externe pour accident domestique, leurs dossiers médicaux ou fiches de suivi doivent comporter obligatoirement la mention “accident domestique” et quelques renseignements spécifiques genre statut socio-économique et niveau d'instruction des parents, statut de la famille monoparentale ou recomposées et effectif etc.; et pour faire participer activement les grands enfants et ses parents à une bonne “sécurisation”, nous recommandant: *Primo*, après chaque consultation même pour un motif autre qu'une lésion par accident domestique, les praticiens doivent donner conseil(court, simple et concis) sur la gravité de ces accidents et les moyens de prévention. *Secundo*, obliger par des textes de loi, les medias et les chaines de télévision d'introduire des pauses éducatives simples, didactiques et de courtes durées au milieu des programmes les plus populaires et les plus regardés que ça soit par les enfants ou les parents.*Tertio*, inclure une attention particulière dans notre contexte de pays islamique à l'inhalation d’épingles à foulard par les jeunes filles qui commencent à porter le voile, afin de réduire son incidence [16]. Mesures qui peuvent aider les praticiens (pédiatres, chirurgiens pédiatres, urgentistes, réanimateurs, généralistes, épidémiologistes…etc.) à bien maitriser la pathologie accidentelle de l'enfant dans ces différents aspects épidémiologique, évolutifs et préventifs.

## Conclusion

Les accidents domestiques de l'enfant constituent rarement une préoccupation de premier plan dans la population, pourtant ils sont parfois très graves et source d'une mortalité importante. Et “pour qu'un accident n'arrive jamais par accident“, il faut en connaître les causes et les circonstances [13]. La prévention de ces accidents est pluridimensionnelle et multidisciplinaire, nécessitant des approches structurelles et organisationnelles, mais aussi informatives et éducatives [10]. Considérés à tort comme inévitables par la population, ces accidents peuvent être évités par la simple application de mesures de prévention active et passive [30, 31]; Faire participer tous les intervenants dans la prévention, ainsi que la multiplication des travaux de recherche épidémiologique (pour le bon chiffrage de ces accidents, de leur mortalité et des séquelles fonctionnelles et psychologiques que peuvent engendrer) constitue la perspective pour éviter les accidents domestiques de l'enfant qui restent potentiellement graves mais toujours sous-estimés.
